# Potato Protein-Based Vegan Burgers Enriched with Different Sources of Iron and Fiber: Nutrition, Sensory Characteristics, and Antioxidants before and after In Vitro Digestion

**DOI:** 10.3390/foods13193060

**Published:** 2024-09-26

**Authors:** Przemysław Łukasz Kowalczewski, Martyna Maria Wróbel, Krzysztof Smarzyński, Joanna Zembrzuska, Mariusz Ślachciński, Paweł Jeżowski, Aneta Tomczak, Bartosz Kulczyński, Magdalena Zielińska-Dawidziak, Karina Sałek, Dominik Kmiecik

**Affiliations:** 1 Department of Food Technology of Plant Origin, Poznań University of Life Sciences, 60-624 Poznań, Poland; dominik.kmiecik@up.poznan.pl; 2 InnPlantFood Research Group, Poznań University of Life Sciences, 60-624 Poznań, Polandpawel.jezowski@put.poznan.pl (P.J.); bartosz.kulczynski@up.poznan.pl (B.K.); 3 Department of Quality Management, Gdynia Maritime University, 81-225 Gdynia, Poland; 4 Institute of Chemistry and Technical Electrochemistry, Poznan University of Technology, 60-965 Poznań, Poland; joanna.zembrzuska@put.poznan.pl (J.Z.); mariusz.slachcinski@put.poznan.pl (M.Ś.); 5 Department of Biochemistry and Food Analysis, Poznań University of Life Sciences, 60-623 Poznań, Poland; aneta.tomczak@up.poznan.pl (A.T.); magdalena.zielinska-dawidziak@up.poznan.pl (M.Z.-D.); 6 Department of Gastronomy Science and Functional Foods, Poznań University of Life Sciences, 60-624 Poznań, Poland; 7 Institute of Biological Chemistry, Biophysics & Bioengineering, School of Engineering & Physical Sciences, Heriot-Watt University, Edinburgh EH14 4AS, UK; k.salek@hw.ac.uk

**Keywords:** plant-based burgers, bioactive compounds, amino acid profile, fatty acid composition, consumer acceptance

## Abstract

The aim of this research was to develop a technology for the production of plant-based burgers (PBBs) based on potato protein, also containing high content of iron and appropriately selected fats. The produced PBBs were characterized in terms of their nutritional and bioactive properties both before and after the in vitro digestion process. It was found that the produced burger was characterized by high protein content, ranging from 20.80 to 22.16 g/100 g. It was also shown to have a high dietary fiber content, ranging from 8.35 to 9.20 g/100 g. The main fraction of dietary fiber in the tested samples was insoluble fiber, which accounted for approximately 89% of the total fiber content. In addition, noteworthy is the high digestibility of the protein, reaching approximately 95% for the potato fiber used in the formulation, and about 85% for the oat fiber. Produced PBBs also provide significant amounts of iron, with the use of an organic iron source greatly increasing its quantity in the final product. The analyzed antioxidant properties before and after the digestion process showed a tenfold increase in biological activity after digestion, indicating that the examined PBBs may counteract oxidative stress. Analyzing the chemical and biological properties, it is impossible not to assess consumer attractiveness. It has been shown that PBB1, which contains potato fiber and powdered sprouts enriched with ferritin, received the highest attractiveness ratings among respondents.

## 1. Introduction

In recent years, we have observed the growing popularity of plant-based diets which has contributed to the dynamic development of the market for food alternatives of animal origin [1]. The takeaway food market, which is also developing dynamically, creates new opportunities and increases the availability of plant-based products, such as vegan burgers offered in catering outlets and for self-preparation at home. Consumers increasingly pay attention to the composition, aroma, and appearance of the products they buy. In the case of meat alternatives, it is crucial that their taste and structure resemble meat as much as possible, which is a challenge in the context of creating wholesome vegan products [2,3].

Modern vegan products are becoming more and more similar to their meat counterparts, both in terms of taste and structure [4,5]. Additives with health-promoting properties are added to them in order to enrich their nutritional value. Animal products are a rich source of amino acids and iron [6,7], however, they also contain cholesterol and saturated fatty acids [8,9]. It is crucial to mention, that vegan burger alternatives can also not be properly balanced, i.e., in terms of amino acids and the content of polyunsaturated fatty acids, such as eicosapentaenoic (EPA), docosahexaenoic (DHA), linoleic (LA), and α-linolenic (ALA). Lack of iron supplementation in vegan diets may lead to increased cases of anemia [10]. However, vegan products can be appropriately enriched with the right ingredients, thus increasing their nutritional value [11,12].

In addition to protein, meat also provides valuable microelements such as iron, zinc, phosphorus, niacin, vitamin B_6_, and vitamin B_12_ [13,14]. High levels of meat consumption, however, lead to an increase in animal agriculture, which contributes to global warming by emitting large amounts of methane [15,16]. Beef production generates as much as 250 times more greenhouse gases than the production of legume protein [17]. Meat production is also a major cause of deforestation, land degradation, water pollution, and desertification [18]. Meat production requires almost 100 times more water than food crops and is also characterized by higher land use, compared to other protein sources. In addition, phosphorus and nitrogen from animal manure pollute surface and ground water, harming water health and human welfare [19,20]. Proteins used to produce meat alternatives have chemical properties similar to animal proteins, which are intended to provide a similar sensory experience. The quality of the protein used in production is crucial from the point of view of human health, because high-quality protein provides the appropriate amount of essential amino acids, which are easily digested and used for protein synthesis [21,22]. The amino acid composition of a protein is the most important factor determining its quality. Food fortification can also be used for plant-based foods, for example by enriching them with “plant iron”—ferritin. Legume seeds are a particularly good source of plant ferritin [23,24]. The consumption of legume seeds has been recommended for years, especially in meatless diets, but this was not intended to be a good source of ferritin. This trend has changed recently and special plant varieties are bred to produce ferritin.

These market dynamics and growing consumer demands create new challenges and opportunities in the context of vegan food production that require further research and innovative technological solutions [1]. Among many plant proteins used in food production, particular attention is drawn to potato protein, which can be obtained from potato juice—an aqueous by-product in the potato starch production process. It is a protein with high biological value—a digestibility of about 70% [25]. This juice contains most of the protein released from the potato tubers when crushed. Moreover, phytocomponents contained in potato juice have a wide spectrum of health-beneficial effects [26]. Importantly, potato protein is characterized by a rich amino acid composition, with a high content of asparagine and leucine compared to other plant proteins, it is one of the most valuable sources of plant protein [25]. Potato proteins have unique physicochemical properties, including the ability to create highly stable foams, which makes them an attractive alternative to chemical additives in the food industry [27]. Therefore, potato protein can be successfully used to produce vegan meat substitutes, constituting a natural alternative to traditional raw materials.

Taking into account the above challenges, the aim of this research was to develop a technology for the production of plant-based burgers (PBBs) based on potato protein, also containing a high content of iron and appropriately selected fats. The obtained PBBs were then characterized in terms of their nutritional and bioactive properties both before and after the in vitro digestion process.

## 2. Materials and Methods

### 2.1. Raw Materials and Reagents

#### 2.1.1. PBBs Ingredients

The potato protein was obtained from potato juice using our patented method based on membrane filtration [28], as previously described [25]. Briefly, fresh potato juice was concentrated via cross-flow ultrafiltration (UF) using a polyethersulfone UF membrane with a 5 kDa cut-off and an area of 3.5 m^2^. The process was conducted at 400 ± 15 kPa, 0.5 m/s cross-flow velocity, and 20 °C. The resulting retentate (potato juice protein concentrate) was spray-dried at 170 °C inlet and 95 °C outlet to ensure stability for long-term storage. The powdered lupine sprouts enriched with ferritin were obtained using a patented [29] technology described earlier [30]. In short, the seeds were disinfected with 70% ethanol for 15 min, then washed and soaked in 0−25 mM FeSO_4_ solutions for 6 h. Germination was conducted for 7 days in FeSO_4_ solutions under controlled conditions. On the final day, seeds were watered and air-dried to 8−10% moisture. Rice protein was purchased from Beneo (Veendam, Belgium), wheat protein from Viresol (Visonta, Hungary), and pea protein from Brenntag (Kędzierzyn-Koźle, Poland). Rice bran oil was purchased from Kasisuri (Bangkok, Thailand), rapeseed oil from ZT Kruszwica (Kruszwica, Poland), and coconut oil from Bazar Zdrowia (Skawa, Poland). Potato starch was obtained from PPZ Trzemeszno (Trzemeszno, Poland) and corn starch from Tar-Groch-Fil (Zakliczyn, Poland). Inactivated yeast flakes were purchased from Naturalnie Zdrowe (Wiązowna, Poland), oat flakes from PW Komplexmłyn (Wągrowiec, Poland), potato fiber from Royal Avebe (Veendam, The Netherlands), oat fiber from ML-Tech (Rutki-Kossaki, Poland), methylcellulose from DuPont (Wilmington, DE, USA), carrageenan from Agnex (Białystok, Poland), and dried beetroot juice from Kaczmarek-Komponenty (Mrowino, Poland). The burger aroma was obtained from Moguntia Food (Suchy Las, Poland). Vinegar and salt were purchased from a local grocery store.

#### 2.1.2. Chemical Reagents and Standards

Folin-Ciocalteu reagent, Na_2_CO_3_, and FeSO_4_ were purchased from Avantor Performance Materials Poland (Gliwice, Poland). TPZT (iron[III]-2,4,6-tripyridyl-S-triazine), ABTS [2,2′-Azino-bis(3-ethylbenzothiazoline-6-sulfonic acid) diammonium salt], pancreatic extract, bile salts, pancreatin, Garche, MRS agar, and MacConkey mediums were purchased from Sigma-Aldrich (Saint Louis, MO, USA). Methanol and acetonitrile LC-MS grade were purchased from Supelco (Merck KGaA, Darmstadt, Germany). *α*-Solanine, *α*-chaconine, and chlorogenic, ferulic, gallic, and caffeic acids were purchased from PhytoLab (Vestenbergsgreuth. Germany). The remaining reagents used for the analyses were purchased from AlfaChem (Lublin, Poland).

### 2.2. PBBs Manufacturing

PBBs were prepared in accordance with the procedure described in Polish patent application No. P.445945 [31]. To structure proteins into powder form, a protein blend was prepared consisting of potato protein (70%), supplemented with rice protein (20%), wheat protein (5%), and pea protein (5%). The blend was mixed with water at a ratio of 1:6 (*w*/*v*) and homogenized using a gastronomic homogenizer (Robot Cook^®^, Robot Coupe, Montceau-les-Mines, France) at 450 rpm for 5 min until a thick suspension was obtained. The suspension was then poured into 250 mL metal molds and baked at 120 °C for 2 h in a Combo oven (MIWE Michael Wenz GmbH, Arnstein, Germany). After baking, the powders were cooled and stored at 4 °C until further use. An oil blend was also prepared using 55% rice bran oil and 45% rapeseed oil to achieve a ratio of n6 to n3 fatty acids of 5:1. For improved structure, coconut oil was additionally used in the burger fillings. Initially, the protein base was mixed with the oils, followed by the addition of dry ingredients. Water and vinegar were then added gradually while continuously mixing to homogenize the filling mass. Subsequently, to equalize moisture and stabilize the filling mass, it was vacuum-packed in low-density polyethylene bags and stored at 4 °C for 24 h. After that time, burgers weighing 75 g each were formed and subjected to thermal processing at 160 °C for 30 min in a convection-steam oven. After cooling, the burgers were vacuum-sealed and stored at 4 °C until analysis. The detailed composition of the analyzed burgers is presented in Table 1.

### 2.3. Nutritional Value and Digestibility

The protein content in the product was determined using the Kjeldahl method according to ISO 1871 [32], using a nitrogen to protein conversion factor of 6.25. The fat content was determined (Soxhlet method) according to the AOAC Official Method 948.22 [33]. The content of soluble fiber (SDF) and insoluble fiber (IDF) was determined using the AACC 32-07 method [34]. The sum of both fiber fractions constitutes the total fiber content. Determination of the moisture was carried out in accordance with the AACC 44-19.01 method [35]. The determination of the total mineral content was carried out according to the international standard ISO 763 [36] using mineralization at a temperature of 550 °C. The carbohydrate content was calculated using the formula:

$$Carbohydrates\left(\%\right)=100-(P+F+Fi+M+W)$$

where: P—percentage of protein, F—percentage of fat, Fi—percentage of fiber, M—percentage of minerals, and W—percentage of water in the analyzed sample. The energy value (EV) was calculated with the following formula:

$$EV\left(\frac{kcal}{100g}\right)=\frac{4\times P+4\times C+9\times F+2\times Fi}{W}$$

The in vitro digestion process and protein digestibility determination were performed according to the method described by Wang et al. [37]. Protein digestibility was calculated based on the difference between the introduced protein and the protein determined in the sample after the in vitro digestion process.

### 2.4. Amino Acids, Fatty Acids, and Mineral Contents

#### 2.4.1. Determination of Amino Acids

The protein hydrolysis was conducted using two distinct methodologies: acidic (110 °C, 23 h) and oxidative (4 °C, 16 h and 100 °C, 2 h), following the official AOAC method 994.12 [38]. Acidic hydrolysis allows for the determination of a wide range of protein amino acids: L-alanine (Ala), L-arginine (Arg), L-aspartic acid (Asp) + L-asparagine (Asn), L-glutamic acid (Glu) + L-glutamine (Gln), L-leucine (Leu), L-lysine (Lys), L-serine (Ser), L-threonine (Thr), L-tyrosine (Tyr), L-valine (Val), L-histidine (His), L-isoleucine (Ile), L-phenylalanine (Phe), L-proline (Pro), and glycine (Gly). Oxidative hydrolysis specifically enables the determination of sulfur-containing amino acids: L-methionine (Met) and L-cystine (Cys). The amino acid contents were subsequently analyzed using the method outlined by Tomczak et al. [39]. Amino acid content is expressed as g/16 g N (equivalent to g/100 g protein).

#### 2.4.2. Determination of Fatty Acid Profiles and Their Nutritional Indices

The fatty acid extraction followed the established protocol outlined by Folch et al. [40]. The analysis of fatty acid composition was conducted according to the AOCS Ce 1 h-05 method [41], with specific parameters detailed in the prior literature [42]. A gas chromatograph (Agilent 7820A, Agilent Technologies, Santa Clara, CA, USA) equipped with a flame ionization detector (FID) and an SLB-IL111 capillary column (100 m length, 0.25 mm diameter, and 0.20 μm film thickness; Supelco, Bellefonte, PA, USA) was utilized for analysis. Results were reported as percentages relative to the total fatty acid content.

Additionally, various nutritional indices related to fatty acids were calculated based on the The ratio of polyunsaturated to saturated fatty acids (PUFA/SFA) was determined to evaluate the influence of diet on cardiovascular health. The atherogenicity index (AI), which reflects the balance between saturated fatty acids (SFA) and unsaturated fatty acids (UFA) in food, was derived using the following formula:

$$AI=\frac{C12:0+\left(4\times C14:0\right)+C16:0}{\sum UFA}$$

Another important measure, the thrombogenicity index (TI), indicates the proportion of pro-thrombogenic SFAs to anti-thrombogenic fatty acids, such as monounsaturated (MUFA), n3, and n6 polyunsaturated fatty acids. This was computed using the formula:

$$TI=\frac{C14:0+C16:0+C18:0}{\left(0.5\times \Sigma MUFA)+(0.5\times \Sigma n6PUFA)+(3\times \Sigma n3PUFA)+(n3/n6\right)}$$

Lastly, the hypocholesterolemic/hypercholesterolemic (HH) ratio, which compares the sum of oleic acid (C18:1) and polyunsaturated fatty acids to the saturated fatty acids from C12:0 to C16:0, was calculated as follows:

$$HH=\frac{cis-C18:1+\Sigma PUFA}{C12:0+C14:0+C16:0}$$

#### 2.4.3. Determination of Mineral Profiles

The determination for minerals (calcium, magnesium, potassium, sodium, copper, iron, manganese, zinc, and lead) was conducted based on the method described by Matusiewicz and Ślachciński [43]. In brief, the freeze-dried material was weighed and placed into a chemically modified Teflon vessel (30 mL), to which 3 mL of concentrated nitric acid and 1 mL of hydrogen peroxide were added. The vessel was then placed in a steel jacket, and for this process a prototype high-pressure/high-temperature system operating in a closed system with concentrated microwave energy was utilized (10 min, 200 W power). Subsequently, the samples were diluted to a volume of 25 mL. Analysis was performed using Inductively Coupled Plasma Optical Emission Spectrometry (ICP OES) with excitation in an Inductively Coupled Plasma (ICP) by an IRIS HR emission spectrometer (Thermo Jarrell Ash, Franklin, MA, USA).

### 2.5. Simulated In Vitro Digestion Process

In vitro digestion was conducted following the method described by Olejnik et al. [44]. Ten grams of lyophilized and ground test material were weighed in glass laboratory bottles, then distilled water was added to achieve a volume of 100 mL. The pH was adjusted to 2.0 using 4 M hydrochloric acid. To simulate stomach processes, pepsin (1.92 mg/g) dissolved in 0.1 M hydrochloric acid was added, and the incubation lasted for 2 h. After incubation, the pH was adjusted to between 7.2 and 7.4 using 1 M sodium bicarbonate to simulate small intestine conditions. To mimic small intestine processes, a solution prepared from pancreatic extract (0.4 mg/g) and bile salts (2.4 mg/g), dissolved in 0.1 M sodium bicarbonate, was added. The sample was incubated for 2.5 h at 37 °C in a shaking water bath. Subsequently, the obtained samples were frozen at −80 °C and then subjected to lyophilization.

### 2.6. Analysis of Changes in Antioxidant Activity Resulting from the Digestion Process

#### 2.6.1. Extraction Process of Antioxidants

The extraction was performed according to the method described by Kowalczewski et al. [45]. The extraction was performed on both the fresh product and the product after digestion, as described in Section 2.5. Briefly, 1 g of the freeze-died sample material was weighed and 9 mL of 80% methanol (*v*/*v*) was added. The mixture was then shaken for 20 min and centrifuged (10,000× *g*, 15 min, 4 °C). The resulting supernatant was filtered through a 0.22 μm PTFE syringe filter.

#### 2.6.2. Total Phenolic Compounds Content Analysis

The analysis was conducted using the Folin-Ciocalteu colorimetric method as described by Singleton et al. [46]. A 4 μL aliquot of the sample was pipetted onto a plate, followed by 16 μL of distilled water and 100 μL of 0.1 N Folin-Ciocalteu reagent, and left to stand for 8 min. Subsequently, 80 μL of sodium carbonate (0.75 g/L) was added and the mixture was incubated at 20 °C for 1.5 h in the dark. Absorbance was measured at 765 nm using a spectrophotometer, and the results were expressed as mg of gallic acid equivalents per gram of dry product weight.

#### 2.6.3. Antioxidant Activity Determined by ABTS Method

The ability to scavenge ABTS radical [2,2′-Azino-bis(3-ethylbenzothiazoline-6-sulfonic acid) diammonium salt] was assessed using the method described by Re et al. [47]. An extract prepared according to Section 2.6.1 was used for the analysis. Briefly, 2 μL of the sample was added to a microplate well containing 200 μL of ABTS radical solution, followed by incubation in the dark at 30 °C for 5 min with continuous shaking. Absorbance was measured using a spectrophotometer at 734 nm wavelength, and the result was expressed as millimoles of Trolox^®^ per gram of dry weight.

#### 2.6.4. FRAP (Ferric Reducing Antioxidant Power) Assay

The ability to reduce iron ions was assessed according to the FRAP method described by Benzie and Strain [48]. An extract prepared following the procedure in Section 2.6.1 was used for the assays. Nine microliters of the sample were pipetted onto a plate, and 270 µL of the TPZT (iron[III]-2,4,6-tripyridyl-S-triazine) working solution were added. The mixture was incubated for 6 min at 36 °C. Spectrophotometric absorbance measurements were taken at a wavelength of 595 nm, and the results were expressed as micromoles of Trolox per gram of dry weight.

### 2.7. Analysis of Changes in the Content of Glycoalkaloids and Phenolic Acids before and after the Digestion Process

#### 2.7.1. Glycoalkaloid Contents

The content of *α*-solanine and *α*-chaconine was analyzed using the method described by Kowalczewski et al. [49] as follows. A 1-g sample of the freeze-dried material was weighed and mixed with 15 mL of 5% acetic acid (*v*/*v*). The mixture was shaken for 15 min and centrifuged (10,000× *g*, 15 min, 4 °C). The supernatant was decanted and subjected to a second extraction under the same conditions. The obtained supernatants were combined. Purification of the samples was achieved using vacuum filtration with an SPE column (HLB Oasis 1 cm^3^ 30 mg, Waters Corporation, Milford, MA, USA). The column was activated sequentially with methanol (2 mL), 10% methanol (*v*/*v*) (2 mL), and distilled water (2 mL). The sample (3 mL) was then loaded and purified with 10% methanol (*v*/*v*) (3 mL). Compounds retained on the column were eluted using methanol with formic acid (0.1% *v*/*v*), and before analysis the samples were filtered through a 0.22 μm PTFE filter. Analysis was conducted using an UltiMate 3000 RSLC chromatographic system (Dionex™, Thermo Scientific Inc., Waltham, MA, USA) coupled with a triple quadrupole API 4000 QTRAP mass spectrometer with electrospray ionization (ESI) (AB Sciex, Foster City, CA, USA) operating in positive ion mode (UHPLC-MS/MS). A Kinetex 1.7 µm C18 column (100 mm × 2.1 mm I.D., Phenomenex Inc., Torrance, CA, USA) was used with a mobile phase consisting of 0.1% formic acid (A) and acetonitrile (B).

#### 2.7.2. Phenolic Acid Contents

The content of phenolic acids including chlorogenic, ferulic, gallic, and caffeic acids was determined using the method described by Cybulska et al. [50], utilizing an extract prepared according to Section 2.6.1. Analysis was conducted using the same LC-MS/MS system employed for the determination of glycoalkaloids. A Luna 3 µm C18 column (150 mm × 2.0 mm I.D., Phenomenex Inc., Torrance, CA, USA) was used with a mobile phase consisting of 5 mM ammonium acetate in water (A) and methanol (B).

### 2.8. Consumer Study

Vegan products are most frequently purchased by a group referred to as “young adults”, which is why consumer attractiveness research was conducted with representatives of this age group. The consumer evaluation of PBBs was analyzed among a group of 100 individuals (age: 19–40 years; women: 67; men: 33) who voluntarily chose to participate in the study. Participants included students and staff from the Poznań University of Life Sciences, who had not undergone specialized training for conducting these tests. Each participant received a consent form and proceeded to the evaluation after giving their consent. The assessment used a hedonic scale ranging from 1 (indicating very low preference) to 9 (indicating very high preference). Various aspects were analyzed, such as taste, aroma, color, texture, appearance, and overall attractiveness. Products were consistently heated to a temperature of ~60–70 °C so that the temperature during the analysis approximated the temperature at the time of consumption.

### 2.9. Statistical Analyses

The experimental data, expressed as mean ± SD, underwent one-way analysis of variance (ANOVA), followed by Tukey’s post hoc test to identify statistically homogeneous groups at α = 0.05 significance level.

## 3. Results and Discussion

### 3.1. Basic Nutritional Value

To characterize the nutritional value of the produced burgers, the content of basic nutrients, including protein, fat, total carbohydrates, and dietary fiber, was determined (Table 2). It was found that the produced burgers were characterized by a high protein content, ranging from 20.80 to 22.16 g/100 g. No statistically significant differences (*p* > 0.05) in protein content were observed between the different burger variants. This is due to the fact that each variant contained the same protein base addition, accounting for 40%. It is worth noting that the protein content in our prepared burgers is similar to that of commercially available vegan burgers, where the protein content typically ranges from about 13 to 27 g/100 g. A similar protein content was also reported by Chilón-Llico et al. [51], who prepared three variants of burgers based on 90% quinoa and 10% lupine, 33.33% quinoa, corn, and lupine, and 50% quinoa and 50% lupine. In these samples, the protein content was 24.6, 19.83, and 18.51 g/100 g, respectively. In another study, De Marchi et al. [11] compared the nutritional composition of meat-based burgers and plant-based burgers currently available in the supermarkets of the European Union. They found that the median protein content in vegan burgers analyzed in the mentioned publication was 18.01%, which is slightly lower than the protein content in our burgers. It is worth noting that the burgers we developed exhibited high protein digestibility, ranging from 84.21% to 95.14%. Lower protein digestibility was observed in PBB2 (85.51%) and PBB4 (84.21%), while higher digestibility was found in PBB1 (95.14%) and PBB3 (95.84%). The observed differences in protein digestibility may result from the type of fiber used in the PBB formulations. PBB1 and PBB3 contain potato fiber, which is characterized by a higher content of insoluble fractions. In contrast, PBB2 and PBB4 use oat fiber, which is rich in soluble fractions. As experts from FAO/WHO/UNU have indicated in a joint report [52], the effects of different sources of fiber vary. They concluded, however, that a reduction in apparent protein digestibility after consuming certain sources of fiber does not necessarily mean that dietary protein is digested and absorbed less well, and the actual difference in digestibility is usually less than 10%. Other studies also suggest that dietary fiber may reduce the digestibility of proteins in food products, with a greater reduction observed in products rich in the soluble fraction of dietary fiber [53,54], which explains the differences we have observed.

Based on the content of individual components (protein, fat, fiber, minerals, and water), the total carbohydrate content was calculated. It was found that the highest total carbohydrate content was in PBB3 (12.34 g/100 g), while the lowest level of carbohydrates was found in PBB2 (7.65 g/100 g) (*p* < 0.05). The differences were most influenced by fiber and mineral content. Determining the content of these components allowed for the calculation of the energy value of the burgers, which ranged from 237.9 to 248.9 kcal/100 g. It should be noted that this value is similar to the energy value of PBBs available on the market in Poland, which usually ranges from 156 to 253 kcal/100 g (data obtained from product labels). Furthermore, Vellinga et al. [55] investigated the energy value of randomly selected PBBs in several European cities (Amsterdam, Copenhagen, Lisbon, and London). They demonstrated that the median energy value of the vegan burgers (233.8 kcal/100 g) was very similar to the caloric content of our burgers.

### 3.2. Amino Acid Profile

The amino acid profile (Table 3) of the proteins present in the produced burgers was analyzed. It was found that the highest proportion of amino acids consisted of glutamic acid and glutamine (18.92–23.24 g/16 g N), which are endogenous amino acids. Samples PBB3 and PBB4 were characterized by higher Glu+Gln content (*p* < 0.05). Additionally, the burgers showed a relatively high content of aspartic acid and asparagine (12.22–14.52 g/16 g N). Among the exogenous amino acids, leucine (9.89–12.11 g/100 g) predominated, which is an important amino acid in human nutrition and is crucial for muscle mass building. For example, Lee et al. demonstrated that leucine supplementation results in increased muscle strength in older individuals with sarcopenia [56]. A similar amino acid profile was reported by De Marchi et al. [11], who showed that the dominant amino acids in plant-based burgers available in the European Union were glutamic acid (median content: 4351.61 mg/100 g of raw material), aspartic acid (median content: 1925.04 mg/100 g), and leucine (median content: 1214.58 mg/100 g).

### 3.3. Fat Content and Fatty Acids Composition

In the prepared burgers, the fat content was also determined (Table 2). The same fat addition (5% coconut oil and 6% oil blend) was applied in each sample. As a result, all burgers exhibited a similar fat content (9.62–10.03 g/100 g), and no statistically significant differences were found between the samples analyzed (*p* > 0.05). When analyzing the nutritional value of commercially available vegan burgers, it can be stated that the burgers developed by us had a relatively low fat content, which in commercial burgers usually ranges from 5.6 to 16 g/100 g. Similarly, in the vegan burgers (90% quinoa and 10% lupine) prepared by Chilón-Llico et al. [51], the fat content was 7.53 g/100 g. However, the remaining variants of the burgers (33.33% quinoa, corn, and lupine; 50% quinoa and lupine) were characterized by significantly lower fat contents, amounting to 5.05 and 4.12 g/100 g, respectively.

No statistically significant differences were observed between the analyzed PBBs in terms of the content of individual fatty acids (Table 4). In the fatty acid profiles of the PPBs, the dominant fatty acids are oleic acid (C18:1, n9), lauric acid (C12:0), and linoleic acid (C18:2, n6). Oleic acid, a monounsaturated fatty acid, is present in the highest concentration, at approximately 30.58% in all samples. Lauric acid constitutes about 21.04% of all fatty acids, and linoleic acid, the dominant polyunsaturated fatty acid, is present at a concentration of around 14.99%. Among the unsaturated acids, oleic acid (C18:1, n9) and linoleic acid (C18:2, n6) are the most abundant. Oleic acid is known for its significant health benefits, particularly in promoting cardiovascular health [57]. The consumption of foods rich in oleic acid and linoleic acid is associated with numerous health benefits, including improved heart health, reduced inflammation, enhanced cognitive function, healthier skin, and a strengthened immune system. Thus, prepared PBBs into the diet can provide a balanced intake of these beneficial fatty acids, contributing to overall health and well-being.

The health value of the oil composition used in the production of PBBs was analyzed (Table 5). As expected, no differences were observed between the samples, as the same oil blend was used throughout. The higher the PUFA to SFA ratio, the more beneficial the oil composition, however, due to the use of coconut oil to achieve the desired structure, the PUFA–SFA ratio remains at around 0.5. The use of coconut oil also affected the AI, TI, and HH indices, lowering the nutritional value of the fat. It is important to note, though, that coconut oil is commonly used in food production, and despite the lower health-promoting fatty acid values in the produced PBBs, these shortcomings can be addressed by following a varied and balanced diet. Since PBBs are only one component of a dish, other ingredients used can compensate for the fat content derived from PBBs.

### 3.4. Dietary Fiber Analysis Results

The analysis of dietary fiber content indicated that the developed burgers are a good source of this nutrient. The highest fiber content was observed in the samples PBB2 (9.20 g/100 g) and PBB4 (9.08 g/100 g), which contained oat fiber (Table 2). A statistically significantly lower (*p* < 0.05) fiber level was found in burgers PBB1 (8.35 g/100 g) and PBB3 (8.42 g/100 g), which included potato fiber. This is most likely due to the fact that, according to the manufacturers’ declarations, oat fiber has a higher fiber content (80 g/100 g) compared to potato fiber (65 g/100 g). It should also be noted that the other ingredients used in burger production (methylcellulose, oat flakes), which were sources of fiber, were added in the same amounts (2% and 4%, respectively) to all burger variants. Based on the conducted studies, it was found that the main fraction of dietary fiber in the tested samples was insoluble fiber, which accounted for approximately 89% of the total fiber content. It is worth noting that, due to such a high fiber content, introducing these burgers to the market would allow the use of the “High fiber” nutrition claim on the product label [58], as they meet the requirements set by the European Parliament and the Council (at least 6 g of fiber per 100 g or at least 3 g of fiber per 100 kcal). The high fiber content in burgers is a highly important aspect in the context of human nutrition. The literature clearly indicates that as many as 90.6% of adolescents, 89.6% of adults, and 83.9% of elderly people do not consume the recommended amounts of fiber (≥30 g/day) [59]. On the other hand, a high intake of dietary fiber has a broad, beneficial impact on health. Conducted studies have confirmed that a fiber-rich diet, among other benefits, significantly increases the population of beneficial bacteria (*Bifidobacterium* and *Lactobacillus*) in the intestines [60], helps regulate blood glucose levels, and reduces the risk of developing type 2 diabetes [61,62].

### 3.5. Minerals

In the produced burgers, a high content of mineral components was found (Table 2). The highest mineral content was observed in PBB3 (7.56 g/100 g), while the lowest level was noted in PBB4 (6.50 mg/100 g). Among the mineral components (Table 6), sodium was present in the highest quantities (14,500–14,900 µg/g dm), with no statistically significant differences between the various burger variants (*p* > 0.05). This is because all burgers had the same salt addition (0.5%). Additionally, a high potassium content was found in all the tested samples (13,200–13,800 µg/g dm). However, it is worth noting that the sodium-to-potassium ratio is rather unfavorable, as it is recommended that this ratio should be less than 0.6 [63]. Our burgers have a ratio of 1.09, but it is possible to modify the recipe to reduce the salt content by using other spices, and thus reduce the sodium content in the burgers. Although this ratio does not apply to individual products but rather to the overall diet, it is worth considering this aspect when developing new food products with high nutritional value. In this case, it is possible to reduce the amount of added salt or increase the addition of ingredients that are good sources of potassium, such as powdered freeze-dried vegetables like parsley, beets, pumpkin, and tomatoes.

It is also noteworthy that De Marchi et al. [11] demonstrated that sodium was the dominant mineral component in commercially available PBBs (median content: 4284.66 mg/kg of the raw product), with potassium being the second most abundant element (median content: 3457.13 mg/100 g). The potassium-to-sodium ratio in these burgers was about 0.8, which was less favorable than in our burgers, where this ratio was around 0.9. Moreover, Vellinga et al. [55] also showed that sodium (388.9 mg) and potassium (220.1 mg/100 g) were among the minerals with the highest levels in PBBs sold in several European cities. Discussing the mineral profile, attention should be paid to the high iron content (385 and 429 µg/g dm) in the PBB1 and PBB2 samples. This is due to the addition of powdered sprouts containing ferritin. Iron was also found in PBB3 and PBB4, to which small amounts of iron (II) sulfate were added. However, the iron content in PBB3 and PBB4 (58.7 and 52.8 µg/g dm) was statistically significantly lower (*p* < 0.05) compared to PBB1 and PBB2. The iron content in the developed burgers is significant because traditional burgers (e.g., beef) are a natural source of this component. Therefore, the addition of iron to vegan burgers allows them, to some extent, to mimic the nutritional value of meat-based burgers. Interestingly, the scientific literature shows that plant-based burgers imitating meat burgers, available in the European Union and the United States, have a higher iron content compared to traditional meat-based burgers [11,64]. This is consistent with our results, as burgers enriched with powdered sprouts containing ferritin or iron (II) sulfate have higher iron levels than what is achievable in meat burgers, which are generally not fortified with this component. In contrast, Latunde-Dada et al. [65] prepared a range of vegan and vegetarian burgers (e.g., based on pumpkin, beetroot, red cabbage, potatoes, and mushrooms) that were not fortified with iron compounds. The iron content in these ranged from 1.13 to 3.39 mg/100 g dry weight. Meanwhile, the iron level in the variant enriched with iron compounds was 14.5 mg/100 g. The same researchers also developed a beef burger recipe containing 95% meat. The iron content in it was 3.42 mg/100 g, which further confirms that plant-based burgers enriched with iron contain higher amounts of iron than traditional meat burgers.

### 3.6. Antioxidant Activity

The Folin-Ciocalteu, ABTS, and FRAP tests performed (Table 7) show that the samples have a relatively low antioxidant potential due to the lack of antioxidants in the burger formulation. Two of the ingredients influencing the antioxidant activity of the tested products are dried beetroot juice and potato proteins. For instance, studies conducted by Mitrevski et al. [66] demonstrated that the addition of beetroot powder to sponge cakes affects the antioxidant properties of the final product. Potato protein is derived from potato juice. Previously published research results indicate a high antioxidant potential of this raw material [67]. In our own research, the presence of phenolic acids (gallic acid, caffeic acid, ferulic acid, and chlorogenic acid) was confirmed in all PBBs, which is also significantly related to the addition of dried beetroot juice and potato protein. The study by Baião et al. [68] confirmed that beets contain the aforementioned phenolic acids, with gallic acid being the predominant one, which is also found in relatively high amounts in our burgers compared to caffeic acid and ferulic acid. At the same time, it should be noted that after the in vitro digestion process, a significant increase in the ability of the burger extracts to scavenge the stable free radical of 2,2′-azinobis-(3-ethylbenzothiazoline-6-sulfonic acid) (50.56–78.58 vs. 3.30–4.72 µmol/g dm) was observed, along with an enhanced ability to reduce iron ions. Additionally, a higher concentration of total phenolic compounds was recorded. Zielinski et al. [69] demonstrated that hydrothermal processing of pasta enhances the levels of all phenolic acids, including both free and ester-bound forms. Similarly, Antoine et al. [70] suggested that *p*-coumaric, salicylic, and ferulic acids, which are covalently attached to plant cell wall polysaccharides, could be released during thermal processing, potentially increasing the total phenolic content (TPC) and the Trolox equivalent antioxidant capacity (TEAC) values as well. It is therefore possible that during the in vitro digestion process, due to the enzymes used and the elevated temperature, bound phenolic acids were released, which is why an increase in the antioxidant activity of PBBs is observed after the digestion process.

### 3.7. Changes in Glycoalcaloids

Due to the use of potato protein in the formulation of PBBs, which, as previously mentioned, is derived from potato juice, it is also necessary to monitor the content of glycoalkaloids. The main glycoalkaloids (GAs) found in potatoes are *α*-solanine and *α*-chaconine [71]. The excessive consumption of GAs can pose health risks to humans, as they have been associated with a range of toxic effects, including gastrointestinal disturbances (such as nausea, vomiting, and diarrhea) and neurological symptoms (such as headaches, dizziness, and confusion). In severe cases, the excessive intake of GAs may lead to more serious health consequences, including respiratory difficulties and even death [72,73]. Therefore, controlling their levels in potato-based products is crucial for ensuring consumer safety. PBB1 and PBB3, in which potato fiber was used in addition to the protein base, were characterized by the highest content of *α*-solanine (Table 8). Similarly, in the case of *α*-chaconine, these variants also contained three to five times more of this glycoalkaloid. GAs are most concentrated in the skin and the area just beneath the skin of the potato tuber. They can also be found in higher amounts in the eyes, sprouts, and any green parts of the potato. Potato fiber is produced from potato pulp, a by-product of potato starch production. The process involves washing and drying the potato pulp, followed by grinding it into a fine powder. For this reason, the use of potato fiber resulted in a significant increase in GAs compared to PBBS produced with the use of oat fiber. Despite the observed similar dependencies in PBBs after the digestion process, i.e., higher GAs content in PBB1 and PBB3, the simulated digestion process significantly reduced the GA content by an order of magnitude in each of the analyzed samples. GAs accumulate in potato tubers as protective factors due to stress and external conditions [74]. Processing tubers with high GA content results in food products with similarly high GA levels because these compounds are heat-resistant and difficult to remove [75]. Due to possible enzymatic and/or acidic hydrolysis upon ingestion, the fate of glycoalkaloids in the gastrointestinal tract is not yet fully understood, and research is ongoing to elucidate the transformations of GAs in the digestive system [76].

It is worth mentioning, however, that low doses of GAs have documented broad health-promoting effects, particularly in the realm of cancer therapy [45,77]. Research has shown that *α*-solanine possesses anti-proliferative properties against various human cancer cell lines, including those from the liver (HepG2), stomach (AGS and KATO III), colon (HT-29), pancreas (Panc-1 and SW1990), lymphoma (U937), leukemia (Jurkat), melanoma (A2058), prostate (PC-3 and DU145), endometrium (RL95-2), esophagus (EC9706), and cervix (HeLa) [77,78,79]. Although α-solanine may be harmful to healthy cells at higher doses, it has the potential to effectively target cancer cells at non-toxic levels [80]. Notably, combining both GAs present in potatoes could result in a synergistic effect [81].

### 3.8. Consumer Acceptance

**Figure 1:**
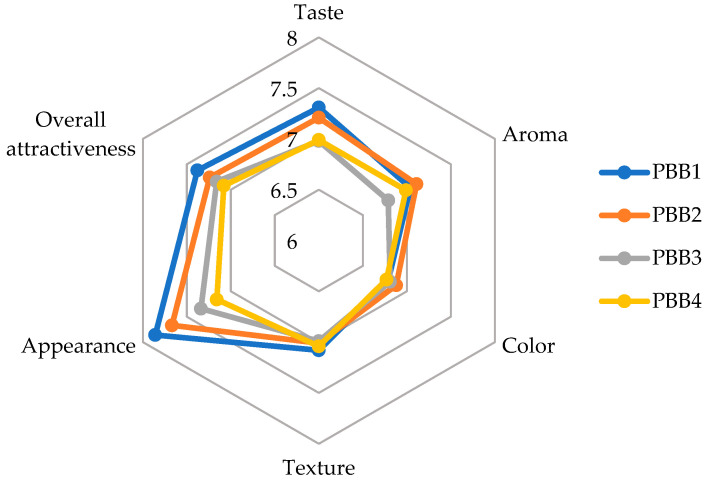
Consumer attractiveness of PBBs.

**Figure 2:**
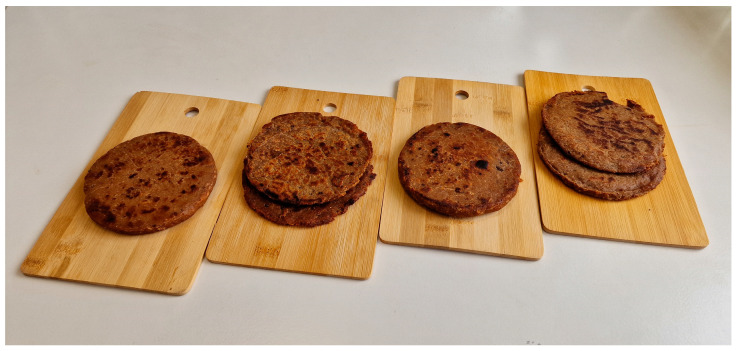
A photograph showing PBBs prepared for consumption. From left: PBB1, PBB2, PBB3, and PBB4.

To succeed in the market, it is not enough to simply use ingredients that enhance the health and nutritional value of products—they must also be appealing and enjoyable for consumers [82,83], therefore, to evaluate the impact of the ingredients used to enrich PBBs, a consumer attractiveness assessment was conducted, the results of which are presented in Figure 1. Analyzing the appearance ratings of PBBS, it was found that PBB1 (presented in Figure 2) received the highest score, followed by PBB2. PBBs containing ferrous (II) sulfate did not receive such high ratings. Similar observations were made for taste and aroma, where PBBs containing ferritin-enriched sprout powder also received higher scores. The sprouts act as a kind of seasoning, enhancing the aroma [84,85]. It is worth noting, however, that both the aroma and taste can be modified by changing the composition of herbs and spices used in the production of PBBs, making this product flexible for the needs of food producers. No differences were observed in the color and texture ratings of the analyzed PBBs. Nevertheless, when analyzing the overall attractiveness, a clear preference for PBB1 is visible, which received the highest ratings.

## 4. Conclusions

Based on the results of the study, it can be concluded that the developed potato protein-based plant burgers are characterized by a high protein content (20.80–22.16 g/100 g) and significant digestibility, particularly in variants containing potato fiber (PBB1 and PBB3), where protein digestibility reached around 95%. The burgers also exhibited a high dietary fiber content, allowing for the use of a “high fiber” claim on product labels. The amino acid profile analysis demonstrated that the PBBs are a good source of essential amino acids, such as leucine, important for muscle mass building, and also contain high levels of endogenous amino acids like glutamic and aspartic acids. The antioxidant properties of the PBBs showed a significant increase in activity following in vitro digestion, suggesting potential health benefits due to the presence of various phytochemicals, such as phenolic acids. Consumer evaluation revealed that PBB1, which includes potato fiber and powdered sprouts enriched with ferritin, received the highest ratings in terms of visual appeal, taste, and aroma. Based on its nutritional and antioxidant properties, as well as consumer acceptance, PBB1 emerges as the most attractive variant of the developed burger analogue. Further studies on the developed PBBs are recommended for demonstrating the potential health benefits arising from the presence of various phytocomponents.

## 5. Patents

The results presented in this article were used to prepare a patent application to the Patent Office of the Republic of Poland (patent application No. P.445945, dated 2023-08-30).

## Figures and Tables

**Table 1:** Recipe compositions of the analyzed PBBs.

Ingredient [%]	PBB1	PBB2	PBB3	PBB4
Protein base	40	40	40	40
Coconut oil	5	5	5	5
Oil blend	6	6	6	6
Potato starch	4	4	4	4
Corn starch	2	2	2	2
Yeast flakes with vitamin B_12_	4	4	4	4
Oat flakes	4	4	4	4
Methylcellulose	2	2	2	2
Carrageenan	2	2	2	2
Aroma	2	2	2	2
Dried beetroot juice	0.75	0.75	0.75	0.75
Salt	0.5	0.5	0.5	0.5
Vinegar	0.5	0.5	0.5	0.5
Potato fiber	2	0	2	0
Oat fiber	0	2	0	2
Powdered sprouts containing ferritin	1.5	1.5	0	0
Iron (II) sulfate	0	0	0.007	0.007
Water	23.75	23.75	25.243	25.243

**Table 2:** Nutrients and energy values of PBBs.

Parameter	Unit	Sample			
		PBB1	PBB2	PBB3	PBB4
Protein content	g/100 g	21.59 ± 0.35 ^a^	21.66 ± 0.86 ^a^	22.16 ± 0.95 ^a^	20.80 ± 1.06 ^a^
Fat content	g/100 g	9.63 ± 0.77 ^a^	9.65 ± 0.60 ^a^	9.62 ± 0.63 ^a^	10.03 ± 0.92 ^a^
Fiber content	g/100 g	8.35 ± 0.18 ^b^	9.20 ± 0.03 ^a^	8.42 ± 0.37 ^b^	9.08 ± 0.11 ^ab^
including:					
IDF	g/100 g	7.45 ± 0.20 ^b^	8.08 ± 0.03 ^a^	7.54 ± 0.39 ^b^	8.05 ± 0.13 ^a^
SDF	g/100 g	0.90 ± 0.03 ^b^	1.12 ± 0.02 ^a^	0.88 ± 0.03 ^b^	1.03 ± 0.05 ^ab^
Carbohydrate content	g/100 g	10.13 ± 0.09 ^b^	7.65 ± 1.52 ^c^	12.34 ± 0.37 ^a^	11.22 ± 1.31 ^ab^
Mineral content	g/100 g	7.10 ± 0.08 ^b^	6.96 ± 0.10 ^b^	7.56 ± 0.07 ^a^	6.50 ± 0.06 ^c^
Energy value	kcal/100 g	248.0 ± 7.5	237.9 ± 2.9	248.9 ± 4.4	245.0 ± 7.2
Protein digestibility	%	95.14 ± 1.66 ^a^	85.51 ± 1.27 ^b^	95.84 ± 1.39 ^a^	84.21 ± 2.01 ^b^

Values marked with the same uppercase letter in the row do not differ significantly *p* > 0.05.

**Table 3:** Amino acid profiles of PBBs.

Amino Acid	Unit	Sample			
		PBB1	PBB2	PBB3	PBB4
His	g/16 gN	3.03 ± 0.12 ^b^	3.48 ± 0.42 ^ab^	4.15 ± 0.70 ^a^	3.97 ± 0.10 ^a^
Ile	g/16 gN	4.93 ± 0.61 ^b^	4.72 ± 0.10 ^c^	5.92 ± 0.69 ^a^	5.38 ± 0.14 ^b^
Leu	g/16 gN	10.15 ± 0.99 ^b^	9.89 ± 0.01 ^c^	12.11 ± 0.48 ^a^	10.98 ± 0.21 ^b^
Lys	g/16 gN	5.67 ± 0.51 ^b^	5.50 ± 0.11 ^b^	6.88 ± 0.37 ^a^	6.35 ± 0.43 ^a^
Met+Cys	g/16 gN	2.99 ± 0.23 ^a^	2.55 ± 0.09 ^b^	2.05 ± 0.42 ^b^	2.67 ± 0.08 ^a^
Phe+Tyr	g/16 gN	7.84 ± 1.11 ^ab^	7.23 ± 0.32 ^b^	8.57 ± 0.24 ^a^	6.72 ± 0.22 ^c^
Thr	g/16 gN	6.11 ± 0.43 ^b^	6.13 ± 0.44 ^b^	6.99 ± 0.28 ^a^	6.54 ± 0.13 ^a^
Val	g/16 gN	5.66 ± 0.58 ^b^	5.56 ± 0.02 ^b^	6.91 ± 0.71 ^a^	6.36 ± 0.18 ^ab^
Ala	g/16 gN	6.75 ± 0.04 ^b^	6.73 ± 0.01 ^b^	8.12 ± 0.60 ^a^	7.76 ± 0.15 ^a^
Arg	g/16 gN	7.41 ± 0.43 ^a^	6.18 ± 0.04 ^b^	7.41 ± 0.53 ^a^	6.12 ± 0.16 ^b^
Asp+Asn	g/16 gN	12.47 ± 0.42 ^b^	12.22 ± 0.09 ^b^	14.52 ± 1.05 ^a^	13.73 ± 1.06 ^a^
Glu+Gln	g/16 gN	19.88 ± 0.20 ^b^	18.92 ± 0.54 ^b^	23.24 ± 1.77 ^a^	21.63 ± 0.91 ^a^
Gly	g/16 gN	8.13 ± 0.73 ^a^	4.21 ± 0.09 ^c^	4.90 ± 0.31 ^b^	4.45 ± 0.05 ^c^
Pro	g/16 gN	5.43 ± 0.23 ^b^	5.23 ± 0.11 ^b^	6.55 ± 0.38 ^a^	5.66 ± 0.15 ^b^
Ser	g/16 gN	6.59 ± 1.41 ^a^	5.66 ± 0.58 ^a^	6.62 ± 0.10 ^a^	6.04 ± 0.21 ^a^

Values marked with the same uppercase letter in the row do not differ significantly *p* > 0.05.

**Table 4:** Fatty acid composition (%).

Fatty Acid	PBB1	PBB2	PBB3	PBB4
C6:0	0.259 ± 0.011	0.261 ± 0.024	0.263 ± 0.009	0.258 ± 0.010
C8:0	3.346 ± 0.037	3.350 ± 0.021	3.347 ± 0.013	3.345 ± 0.011
C10:0	2.655 ± 0.008	2.653 ± 0.006	2.648 ± 0.010	2.651 ± 0.009
C12:0	21.043 ± 0.011	21.033 ± 0.007	21.029 ± 0.006	21.052 ± 0.010
C14:0	8.409 ± 0.006	8.422 ± 0.004	8.401 ± 0.007	8.416 ± 0.009
C16:0	11.137 ± 0.012	11.133 ± 0.022	11.127 ± 0.016	11.140 ± 0.014
C16:1	0.109 ± 0.003	0.106 ± 0.004	0.110 ± 0.003	0.102 ± 0.006
C18:0	2.335 ± 0.006	2.341 ± 0.008	2.329 ± 0.006	2.334 ± 0.005
C18:1 (n9)	30.583 ± 0.018	30.591 ± 0.026	30.576 ± 0.012	30.587 ± 0.010
C18:2 (n6)	14.991 ± 0.012	14.983 ± 0.020	14.996 ± 0.019	14.988 ± 0.011
C18:3 (n3)	2.286 ± 0.012	2.281 ± 0.023	2.279 ± 0.018	2.292 ± 0.014
C18:3 (n6)	0.116 ± 0.004	0.112 ± 0.008	0.120 ± 0.008	0.111 ± 0.009
C20:0	0.381 ± 0.006	0.383 ± 0.004	0.377 ± 0.009	0.392 ± 0.010
C20:1	0.445 ± 0.008	0.451 ± 0.019	0.416 ± 0.032	0.444 ± 0.022
C22:0	0.146 ± 0.002	0.144 ± 0.006	0.144 ± 0.008	0.150 ± 0.008
C22:1	0.056 ± 0.002	0.061 ± 0.002	0.058 ± 0.003	0.052 ± 0.001
∑SFA	49.71	49.72	49.66	49.74
∑MUFA	31.19	31.21	31.16	31.18
∑PUFA	19.10	19.07	19.18	19.08

SFA—Saturated Fatty Acids; MUFA—Monounsaturated Fatty Acids; PUFA—Polyunsaturated Fatty Acids.

**Table 5:** Values of the nutritional index used to evaluate the quality of the oil blends’ nutrition.

Sample	PUFA/SFA	AI	TI	HH
PBB1	0.35	1.35	0.73	1.18
PBB2	0.35	1.35	0.73	1.18
PBB3	0.35	1.35	0.73	1.18
PBB4	0.35	1.35	0.73	1.18

**Table 6:** Mineral profiles of PBBs.

Parameter	Unit	Sample			
		PBB1	PBB2	PBB3	PBB4
Ca	µg/g dm	1240 ± 100 ^a^	1210 ± 100 ^a^	1250 ± 100 ^a^	1150 ± 90 ^a^
Mg	µg/g dm	508 ± 41 ^a^	566 ± 45 ^a^	490 ± 39 ^a^	492 ± 39 ^a^
Na	µg/g dm	14,500 ± 1100 ^a^	14,500 ± 1100 ^a^	14,800 ± 1200 ^a^	14,900 ± 1200 ^a^
K	µg/g dm	13,400 ± 1100 ^a^	13,600 ± 1100 ^a^	13,200 ± 1100 ^a^	13,800 ± 1100 ^a^
Fe	µg/g dm	385 ± 31 ^b^	429 ± 34 ^a^	58.7 ± 4.7 ^c^	52.8 ± 4.2 ^c^
Zn	µg/g dm	54.8 ± 4.4 ^a^	55.6 ± 4.4 ^a^	54.9 ± 4.4 ^a^	52.2 ± 4.2 ^a^
Cu	µg/g dm	54.4 ± 4.4 ^a^	49.7 ± 4.0 ^a^	40.3 ± 3.2 ^b^	40.2 ± 3.2 ^b^
Mn	µg/g dm	47.3 ± 1.4 ^b^	47.9 ± 3.8 ^b^	49.4 ± 4.0 ^a^	49.6 ± 4.0 ^a^
Pb	µg/g dm	94.4 ± 7.6 ^a^	81.8 ± 6.5 ^a^	56.7 ± 4.5 ^b^	56.6 ± 4.5 ^b^
Cd	µg/g dm	0.957 ± 0.068 ^b^	1.112 ± 0.143 ^ab^	1.061 ± 0.101 ^b^	1.296 ± 0.300 ^a^

Values marked with the same uppercase letter in the row do not differ significantly *p* > 0.05.

**Table 7:** Antioxidants and antioxidant capacity before and after digestion.

Parameter	Unit	Sample			
		PBB1	PBB2	PBB3	PBB4
Before digestion process					
TPC	mg/g dm	0.380 ± 0.082 ^a^	0.343 ± 0.024 ^a^	0.167 ± 0.020 ^b^	0.380 ± 0.249 ^a^
TEAC_ABTS_	µmol/g dm	3.305 ± 0.653 ^b^	4.726 ± 0.046 ^a^	3.408 ± 0.455 ^b^	4.621 ± 0.446 ^a^
TEAC_FRAP_	µmol/g dm	1.230 ± 0.043 ^b^	1.466 ± 0.059 ^a^	0.823 ± 0.040 ^c^	1.275 ± 0.048 ^b^
Gallic acid	µg/g dm	2.464 ± 0.068 ^b^	2.658 ± 0.386 ^a^	2.501 ± 0.001 ^b^	2.548 ± 0.033 ^ab^
Caffeic acid	µg/g dm	0.952 ± 0.063 ^a^	0.402 ± 0.021 ^b^	0.964 ± 0.044 ^a^	0.438 ± 0.019 ^b^
Ferulic acid	µg/g dm	1.210 ± 0.044 ^a^	1.085 ± 0.071 ^b^	1.175 ± 0.100 ^a^	1.025 ± 0.054 ^b^
Chlorogenic acid	µg/g dm	3.315 ± 0.064 ^a^	2.781 ± 0.154 ^b^	3.216 ± 0.150 ^a^	3.107 ± 0.105 ^ab^
After digestion process					
TPC	mg/g dm	4.016 ± 0.092 ^a^	3.616 ± 0.177 ^b^	3.687 ± 0.369 ^ab^	4.141 ± 0.202 ^a^
TEAC_ABTS_	µmol/g dm	50.56 ± 6.78 ^b^	75.56 ± 9.16 ^a^	51.39 ± 10.17 ^b^	78.58 ± 7.26 ^a^
TEAC_FRAP_	µmol/g dm	2.296 ± 0.301 ^a^	1.875 ± 0.342 ^b^	1.751 ± 0.098 ^b^	2.293 ± 0.204 ^a^
Gallic acid	µg/g dm	1.541 ± 0.161 ^a^	1.601 ± 0.082 ^a^	1.653 ± 0.019 ^a^	1.640 ± 0.063 ^a^
Caffeic acid	µg/g dm	0.116 ± 0.017 ^a^	0.123 ± 0.011 ^a^	0.118 ± 0.007 ^a^	0.123 ± 0.003 ^a^
Ferulic acid	µg/g dm	0.938 ± 0.031 ^a^	0.811 ± 0.010 ^b^	0.894 ± 0.131 ^a^	0.816 ± 0.010 ^b^
Chlorogenic acid	µg/g dm	0.697 ± 0.093 ^a^	0.804 ± 0.043 ^a^	0.744 ± 0.001 ^a^	0.749 ± 0.035 ^a^

Values marked with the same uppercase letter in the row do not differ significantly *p* > 0.05.

**Table 8:** Glycoalcaloid content in PBBs before and after digestion.

Parameter	Unit	Sample			
		PBB1	PBB2	PBB3	PBB4
Before digestion process					
*α*-solanine content	µg/g dm	40.69 ± 2.68 ^a^	28.74 ± 3.29 ^b^	39.57 ± 0.22 ^a^	22.28 ± 11.56 ^c^
*α*-chaconine content	µg/g dm	15.11 ± 2.49 ^a^	5.09 ± 0.57 ^b^	15.78 ± 0.43 ^a^	3.48 ± 0.96 ^c^
After digestion process					
*α*-solanine content	µg/g dm	3.43 ± 0.57 ^bc^	2.59 ± 0.17 ^c^	5.97 ± 0.44 ^a^	3.66 ± 0.77 ^b^
*α*-chaconine content	µg/g dm	1.13 ± 0.15 ^b^	0.37 ± 0.06 ^c^	2.10 ± 0.06 ^a^	0.53 ± 0.18 ^c^

Values marked with the same uppercase letter in the row do not differ significantly *p* > 0.05.

## Data Availability

The original contributions presented in the study are included in the article; further inquiries can be directed to the corresponding authors.
